# Building the Workforce’s Capacity to Support the Digital Transformation of Public Health: Environmental Scan of Training Programs for Digital Technologies in Public Health

**DOI:** 10.2196/73088

**Published:** 2025-10-15

**Authors:** Ihoghosa Iyamu, Swathi Ramachandran, Hsiu-Ju Chang, Andre Kushniruk, Francisco Ibáñez-Carrasco, Catherine Worthington, Hugh Davies, Geoffrey McKee, Adalsteinn Brown, Mark Gilbert

**Affiliations:** 1 School of Population and Public Health, University of British Columbia, Vancouver, BC, Canada; 2 BC Centre for Disease Control, 655 West 12th Avenue, Vancouver, BC, V5Z4R4, Canada, 1 6047075619; 3 School of Health Information Science, University of Victoria, Victoria, BC, Canada; 4 Dalla Lana School of Public Health, University of Toronto, Toronto, ON, Canada; 5 School of Public Health and Social Policy, University of Victoria, Victoria, BC, Canada

**Keywords:** digital health technology, public health schools, professional competence, public health education for professionals, digital health, competency-based education, health workforce

## Abstract

**Background:**

The digital transformation of society and public health has created an urgent need for new competencies to address evolving and contemporary public health challenges. While some public health institutions and schools worldwide have begun responding through various training programs and approaches, many have yet to do so. A clearer understanding of the current training landscape can inform more coordinated efforts to update curricula and strengthen digital competency within the public health workforce.

**Objective:**

This study aimed to map and describe existing digital public health (DPH) training programs, identifying common curricula content, disciplinary involvement, and training approaches. It also aimed to identify gaps and opportunities for curricular adaptation.

**Methods:**

This environmental scan was conducted in 2 stages, drawing on guidance from studies by Rowel et al and Wilburn et al. First, we performed a systematic search of DPH training programs, followed by interviews with selected program directors to explore their program design and implementation. The scan emphasized a transdisciplinary lens, consistent with the evolving nature of DPH. Between March and May 2023, we searched Google and public health association directories to identify degree programs and courses (as part of degree awarding programs) focused on building capacity for using digital technologies in public health. We then conducted semi-structured interviews with 4 directors of identified programs exploring program characteristics and the inter- or transdisciplinary partnerships essential to their design. Search data were summarized using narrative synthesis, while content analysis was applied to the interview data.

**Results:**

Overall, 58 DPH training programs were identified, categorized into 3 groups: public health data science (29/58, 50%), public health informatics (16/58, 28%), and a mix of programs exploring digital competencies (13/58, 22%) related to project management and addressing the digital determinants of health. Interviews revealed that motivation for developing interdisciplinary DPH programs stemmed from the need to meet evolving job market demands and respond to calls for curricular renewal among professional bodies. Effective design and delivery were supported by academic–industry partnerships, which aimed to cultivate professionals with depth in public health and breadth in digital competencies. These programs drew on diverse disciplinary perspectives from academia, the public sector, and private industry. However, sustaining such partnerships was challenged by the need to negotiate shared priorities, reconcile differing viewpoints, and secure ongoing funding.

**Conclusions:**

This global scan of DPH training programs found a strong focus on data-centric competencies, with less emphasis on digital skills for health promotion, leadership, and addressing digital determinants of health. Bridging these gaps requires a stepwise approach: integrating digital competencies into existing curricula, offering stand-alone programs for specialized skills, and strengthening partnerships to navigate funding and administrative barriers while promoting equity-driven, interdisciplinary collaboration.

## Introduction

The digital transformation of society, including public health, was significantly accelerated during the COVID-19 pandemic [1-3]. Digital technologies have been adopted to enhance the efficiency, effectiveness, and reach of essential public health functions, such as real-time precision disease surveillance using big data and artificial intelligence, targeted health promotion via social media, apps, and wearables, and environmental health protection informed by sensor data [2-5]. Digital access and literacy have recently emerged as important determinants of health, directly and indirectly shaping public health outcomes [6-8]. For example, limited digital access and literacy may restrict social and economic opportunities, which in turn limit access to digitally mediated health and social services [8,9]. Conversely, for those with digital access, exposure to misinformation, disinformation, and exploitative practices by big tech platforms can adversely impact health and health behaviors [8,10,11].

To harness these opportunities and address the evolving challenges, the contemporary public health workforce must develop new digital competencies [12,13]. In our previous research, we conducted a rapid literature review and identified 45 unique digital competency statements that cut across all 7 categories of the 2008 Core Competencies for Public Health in Canada framework [12]. We also identified a potentially new competency category related to digital data, data systems management, and governance [12]. While these competencies are not exhaustive, especially with rapidly developing digital technologies, they represent aspirations of public health practitioners and educators to address existing competency and curricular gaps [14,15]. For instance, the Association of Schools for Public Health in the European Region (ASPHER) task force for digital transformation has recommended updates to the public health curriculum across themes such as digital literacy, health data collection and analysis, health data management and governance, ethics and regulation of digital transformations in society, and understanding the infosphere and spread of information over digital networks [16]. They emphasize the need for interdisciplinary and transdisciplinary academic communities that understand public health, as well as computer science, data science, and other sciences relevant for digital public health (DPH) [16,17]. Ongoing efforts by the Public Health Agency of Canada, including commissioning the National Collaborating Centers for Public Health to update the Core Competencies for Public Health in Canada, are also examples of work to address these competency needs [18].

It remains unclear how these competency aspirations and curricular recommendations have been implemented in public health training programs and courses. Gaining insight into how these competencies have shaped curricular updates can guide targeted recommendations for digital competency development and curriculum design in Canada. This would support the broader vision of transforming public health and enable schools of public health to make necessary adaptations [13]. In addition, with well-documented challenges in inter- and transdisciplinary practice, there is limited understanding of the disciplinary perspectives incorporated into these programs and how educators navigate diverse viewpoints in practice [17,19].

Therefore, our main objective was to map, explore, and describe training programs designed to integrate and enhance the use of digital technologies specifically for public health and its subspecialties around the world. Specifically, we examined common curricular content, the academic disciplines involved in program design and delivery, and the training approaches used to implement these curricula. We also identified opportunities for curriculum adaptation and strategic pivots in response to emerging needs and gaps in current DPH curricula and training programs. This work is part of a broader study supporting the digital transformation of public health by informing competency development, encouraging interdisciplinary collaboration, and guiding future curriculum planning [12,20]. Ultimately, this work seeks to better prepare public health practitioners to engage with and lead within an increasingly digital health landscape.

## Methods

### Study Design

The study was an environmental scan conducted following a 2-stage approach: a systematic search for DPH programs aligned with the study objectives and semistructured interviews, with program directors or faculty members from programs emphasizing inter- or transdisciplinary perspectives in their curriculum planning and delivery [21,22].

### Systematic Search for DPH Program Websites

Two members of the research team (II and SR) conducted independent searches using Google’s advanced search (google.com/advanced_search) without country restrictions. Search terms included (“digital public health” OR “digital health” OR “digitalization” OR “digital transformation” OR “data science” OR “social media” OR “artificial intelligence” OR “machine learning”) AND (“school of public health” OR “college of public health” OR “department of public health” OR “division of public health” OR “institute of public health”) AND (degree OR program OR course). Thereafter, the search was repeated, restricting returns to countries identified as prominent contributors to DPH literature, including Australia, Canada, China, France, Germany, Italy, Sweden, the United Kingdom, and the United States of America (Multimedia Appendix 1) [1,17]. We reviewed the first 100 returns for each search. Thereafter, we searched popular global online directories of schools of public health, including the Council on Education for Public Health, ASPHER, and the Public Health Agency of Canada, to identify potential programs or courses for review. All relevant returns were reviewed against the inclusion and exclusion criteria (Table 1). All searches were conducted between March 24, 2023, and May 16, 2023.

**Table 1.** Program inclusion and exclusion —environmental scan of digital public health training programs (2023).

Domain	Inclusion criteria	Exclusion criteria
Population	Focus on public health students, public health informaticians and analysts, practitioners (including public health physicians), public health researchers, and decision- and policy-makers.	Exclusive focus on clinicians, programmers, or other related technologists, informaticians, or other allied health system practitioners.
Concept	Programs focused on building capacity for designing, implementing, and evaluating digital technologies in any of the domains of public health or across public health.	Programs focused on the broader field of digital health or a mostly clinical perspective (eg, telemedicine or virtual health in a clinical context).
Context of training program (ie, context of application)	Population and public health	Clinical or solely clinical contexts
Program type	Established degree awarding training programs at undergraduate and graduate levels or courses undertaken as a requirement for a degree awarding public health program.	Short courses, workshops, webinars, and courses not related to a public health program.
Timeline	Must be currently available for enrollment	Previously available programs that have been discontinued or truncated.

### Program Selection

We adopted a definition of an eligible DPH training program as any undergraduate or graduate-level degree-awarding public health training programs that were currently available for enrollment and entirely focused on applying digital technologies to all or to specific public health domains as defined in the Canadian Public Health Association’s conceptual framework for public health (Table 1) [23]. We also included course modules that are part of degree-granting public health programs. In addition, programs with a broader medical focus were included if they explicitly addressed public health, such as through modules relevant to public health as described within their overall objectives. We excluded programs and courses that are exclusively focused on clinical and clinical informatics perspectives and those offered as short courses (ie, stand-alone course modules not offered as part of a degree-awarding program). One member of the research team (SR) reviewed search returns based on our inclusion and exclusion criteria (Table 1), with another researcher (II) reviewing the list of included programs and courses for accuracy based on the inclusion and exclusion criteria.

### Data Collection

We reviewed the websites for each included training program and course, identifying program documents and training curricula available on their websites. Data from these documents were extracted using pretested data extraction forms (Multimedia Appendix 2) adapted from the guidelines for reporting evidence-based practice educational interventions and teaching [24]. Extracted data included basic program information (eg, program name, country, city, and year of commencement) and program descriptors (eg, target audience, level of training, educational materials used, characteristics of the instructor(s), and underpinning theories ([ie, educational or field-specific theory(ies) guiding the program]). For program websites not originally in English, Google Translate was used to access the documents before extraction.

### Semi-Structured Interviews

We reviewed online documents for each included program to identify evidence of a clear mandate for inter- and transdisciplinary training. This included mission statements, faculty from diverse disciplines, and explicitly stated inter- or transdisciplinary partnerships. For each qualifying program, we invited program directors or staff involved in program design, delivery, and evaluation to participate in semi-structured interviews. Additional eligibility criteria included being an established graduate- or undergraduate-level degree-awarding program with English-speaking staff. Recruitment emails were sent using contact details from program websites, and a convenience sample of staff who responded to invitations was recruited. Each eligible program received up to 3 reminders. Using a snowball sampling approach, we identified an additional program that was not on the original list, as it did not have a listed degree-awarding program on its website. However, their strong emphasis on transdisciplinary DPH training and traditional public health training focused on DPH research justified their inclusion in the interviews. Interviews were conducted with consenting participants over Zoom Online (Zoom Communications Inc.). We used a topic guide that explored characteristics of the DPH training programs, academic and practice disciplines involved in designing and delivering the program curricula, characteristics of these inter-/transdisciplinary collaborations, and lessons learned (Multimedia Appendix 3). The topic guide was developed based on initial findings from our rapid review of DPH competency recommendations, insights from previous DPH-related literature reviews, and consultations with education leads from schools of public health who served as co-investigators on the project [12,17]. All interviews were conducted by II, who is trained in qualitative health research methods, supported by SR who received training on the data collection protocol and made detailed field notes. All interviews were audio recorded and automatically transcribed using Zoom’s auto-transcribe function. Transcripts were reviewed for accuracy and corrected as appropriate. Data analyses were concurrently conducted alongside interviews. Each interview lasted on average 47 minutes (range 39‐53 min).

### Ethical Considerations

This study was reviewed by the University of British Columbia’s Behavioral Research Ethics Board (ethics #H22-03153). We obtained written informed consent from each participant at least 24 hours before the interviews via email and assigned each participant an identification number, which was included in all interview transcripts and field notes (Multimedia Appendix 4). All study materials were stripped of personal identifiers before analyses. Participants did not receive an honorarium.

### Data Analyses

We summarized data from the systematic search using descriptive statistics (frequencies and proportions) in Microsoft Excel and conducted a narrative synthesis. For the semistructured interviews, transcripts and field notes were imported into QSR NVivo version 1.3 for data management and analysis [25]. We conducted a content analysis exploring key descriptions of programs, inter/transdisciplinary collaborations, and lessons learned from implementing such collaborations within the program context. Our analyses followed an inductive process beginning with initial coding, categorization, and interpretation of findings [26,27]. Emergent codes and categories were reviewed with the research team consisting of public health educators and DPH experts whose views informed ongoing refinement and interpretation of codes and categories generated during analysis [27]. Throughout the analyses, II and SR made reflexive memos about our perspectives of the data [28]. These memos also informed the analysis and the final report draft.

## Results

### DPH Programs and Courses Identified From Systematic Search

#### Overview of Included Programs

**Figure 1.**
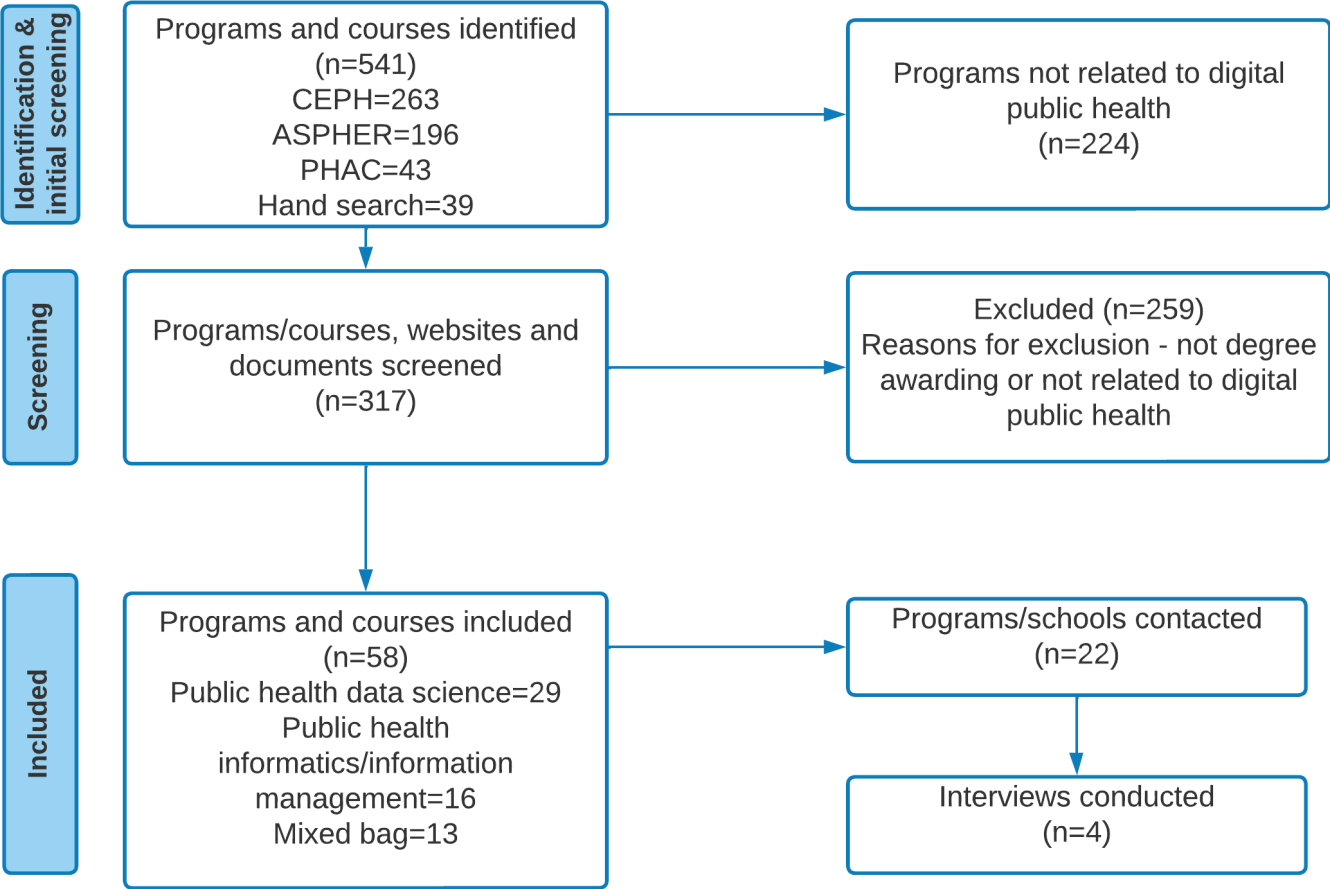
Flow diagram of the program search and selection process.

Overall, we identified 58 DPH training programs and courses offered as requirements for a degree awarding program applying digital technologies for public health functions (Figure 1. Of 58 programs, 35 (60.3%) were from universities and colleges in the United States (Table 2), 46 (79.3%) were at the master’s degree level, 25 (43.1%) were delivered in-person (including options for applied components such as practicums and internships), and 47 (81%) did not explicitly describe theoretical foundations of their programs and courses (Multimedia Appendix 5). Regarding foundational theories underpinning programs, only 11 (18.9%) of 58 explicitly described being built on modern statistical theories, including probability and Bayesian frameworks.

Identified programs focused on 3 main DPH categories: public health data science (29/58, 50%) programs focused on using modern statistical and analytic approaches to harness digital data streams to provide evidence for public health decision making; public health informatics (16/58, 28%) programs focused on using information, computer science, and technology to create and manage the infrastructure and systems needed for digital data streams and analytic techniques; and a mix of programs and courses (13/58, 22%) focused on various digital competencies, including digital leadership, project management, digital health communication, digital transformation leadership, digital design and implementation, digital monitoring and evaluation, and tackling the digital determinants of health. There was significant overlap between programs across all 3 categories. However, the first 2 focused on a previously identified competency category related to digital data, data systems management, and governance.

**Table 2.** Characteristics of included DPH training programs and mandatory courses—environmental scan (2023).

Program characteristics	Frequency (N=58), n (%)
Country of program	
USA	35 (60.3)
Canada	6 (0.3)
UK	4 (6.9)
Australia	2 (3.4)
Germany	2 (3.4)
India	2 (3.4)
Others	7 (2.1)
Level of training	
Undergraduate	4 (6.9)
Master’s	46 (79.3)
PhD (Doctoral level)	1 (1.7)
Combined Master’s and Doctoral training	4 (6.9)
Others	3 (5.2)
Foundational theories	
Not stated	47 (81.0)
Biostatistics/statistics, probability, or related theories	10 (17.2)
People centeredness^a^	1 (1.7)
Partnerships reported (ie, public and private sector partners)	
Not stated	51 (87.9)
Industry partners (eg, health departments and health analytics consulting groups)	4 (6.9)
Health industry and university partnerships	3 (5.2)
Delivery	
Not stated	12 (20.7)
Online^b^	9 (15.5)
Blended	12 (20.7)
In person	25 (43.1)

a Emphasizes person-centredness as an organizing framework, in contrast to traditional informatics approaches.

b Includes online-delivered programs that incorporate in-person practicums or internships.

#### Curricula Content and Training Approaches

Identified programs had diverse learning objectives, foundational components, and emphasized various public health domains and functions. The majority of the public health data science programs and courses explored subjects related to public health sciences, biostatistics, computer science, and variants of these core content areas as presented as data science training. Most of these programs focused on public health and health services research, epidemiology, disease surveillance, and prevention. Public health informatics programs leveraged information science, computer science, data management, and health management information systems as applied to public health generally. The mixed bag of programs and courses leveraged varied digital content applied in health promotion, program development and evaluation, epidemiology, disease surveillance, and public health more generally. The majority of the programs (33/58) were delivered online, while others used blended learning (12/58) or in-person training (12/58). Most of the in-person training involved a blend of coursework and internships or practicums in real-world settings. The majority of these were focused on public health data science and public health informatics. Importantly, courses within the mixed-bag category leveraged industry–academic partnerships to facilitate content delivery as appropriate.

#### Disciplines Involved in Training Programs

While most programs highlighted their multi-, inter-, or transdisciplinary perspectives using broad statements, 17/58 programs (29.3%) did not clearly describe the disciplinary perspectives involved in the design and delivery of the training programs and courses. Where specified, programs predominantly indicated specific disciplines, including public health sciences (including specific fields such as epidemiology and biostatistics). Beyond public health sciences, programs highlighted the involvement of statistics, computer science, information technology and informatics, health care administration, mathematics, life and health sciences, media and communication, health economics, law bioethics, psychology, anthropology, management, and so on (Multimedia Appendix 5).

### Qualitative Interview Results—Considerations for Interdisciplinarity

#### Overview

A total of 4 program directors or faculty representing 4 of the included programs participated in interviews. Interviews were with program directors of programs in Canada, Australia, Germany, and the Netherlands. From the interviews, we identified 4 main qualitative categories that characterize interdisciplinary partnerships and offer insights into implementing transdisciplinary DPH programs in real-world settings. Each category is supported by exemplar quotes.

#### Motivation For Interdisciplinary DPH Programs

This reflected program directors’ impetus for developing programs focused on interdisciplinarity. First, programs recognize the need to align their curricula and training with evolving digital trends in public health, responding to job market competency requirements and current course offering gaps based on previous research and professional experiences that emphasize the need for applied skills in inter- and transdisciplinary settings. Moreover, program directors’ interdisciplinary training was also a key motivation for developing and implementing interdisciplinary programs that reflect their understanding of the current job market. For example, one participant said:

*I am pure technologist by training, and being a digital health researcher, my research findings suggested that there is a need to have consumer education in public health... So, I wouldn't say that I'm an expert in public health that’s not my area of expertise, but I've learned a lot in the past few years since I've been here*...

[Participant 2]

Program directors emphasized the need to align their programs with curricular updates from professional bodies (such as ASPHER and the Australian Digital Health Agency) that have issued recommendations for digital competencies. They also noted that interdisciplinary programs were designed to address students’ expressed learning needs, emphasizing the importance of experiences that closely reflect real-world working conditions.

#### Design and Delivery of Interdisciplinary Programs

Program directors primarily designed their programs with a focus on desired student outcomes. A common aspiration was to develop *T-shaped professionals*—individuals with broad knowledge across digital technology fields and deep expertise in at least one public health discipline. For most programs, their design was informed by active partnerships with industry practitioners, including private sector partners. For example, some program directors described program design supervised by an advisory board consisting of public and private industry partners that highlighted competencies needed in the workplace. Programs were structured around competencies aimed at fostering interdisciplinary collaboration, as well as practical understanding of digital regulatory frameworks, leadership in digital technology, design thinking, and advanced analytics.

While directors acknowledged the limited availability of competency frameworks in this area, they often described building curricula based on their vision for student outcomes. This involved inviting key partners to contribute and leveraging their various disciplinary expertise areas to align with the program’s goals:

*I think what really helped us work together was having this clear vision of you know this is who the ideal graduate is. This is what we want them to be able to do in the workplace, and then taking that vision and really breaking it apart, right? And then the data scientists could really say, okay, if I put myself in a position of having to work with this person, what would I want them to know? And I could put myself in the position of okay. If this is a person that’s going to advise me on governance issues, you know, hospital policy, these kinds of things. What would I want them to tell me about technology, right? ... So, that allowed us to again retreat in our, you know, disciplinary silos. But at least work on the curriculum as a team, right? But again, it was from our perspectives*...

[Participant 1]

Regarding training approaches, all interviewed programs described prioritizing experiential learning using a combination of foundational class-based courses and practicums or internships within public health organizations demonstrating interdisciplinary work. For instance, one of the public health data science programs is implemented with triads within the program, combining students of varied backgrounds (eg, public health sciences, computer science, and biostatistics) to solve real-world problems within health systems.

#### Characteristics of Interdisciplinary and Transdisciplinary Partnerships Supporting Identified Programs

Program directors suggested that interdisciplinary partnerships are built off a core group of leaders who can engage other disciplines based on the goals for the “T-shaped professional.” Partnerships span multiple disciplines, including public health and medicine (eg, biostatistics, epidemiology, public health sciences, clinical medicine, psychology); applied sciences and technology (eg, computer science, data science, human–computer interaction, informatics, and cybersecurity); and the arts and humanities (eg, law, bioethics, media and communications, and anthropology). These partnerships essentially were prioritized based on the competencies for the envisioned T-shaped professional, starting within the internal academic environment first, extending to public and private sector partners as needed, including the formation of advisory boards as described. In addition, these partnerships are leveraged for curricular content delivery to align student experiences with real-world experiences and expectations:

*We have a what we called a stakeholder advisory board as well where we have some people from industry and we had different workshops where we invited people to present you know what they had been doing what they're forward or line of thinking is* ...

[Participant 4]

#### Challenges With Implementing Interdisciplinary and Transdisciplinary Partnerships

Program directors highlighted a range of challenges associated with establishing transdisciplinary partnerships, as well as those stemming from administrative processes or the implementation of innovative programs within established schools. In terms of partnerships, challenges included negotiating shared commitment and perspectives among diverse experts with differing methodologies, views on knowledge, and levels of commitment to public health principles. These issues were particularly pronounced in academic–industry collaborations, especially with private entities. Concerns centered on the potential for noncritical, techno-optimistic implementation of technologies and the risk of exploitation within such partnerships. Participants emphasized the importance of having a clear understanding of the principles driving the operations of private entities. For example, one participant stated:

*I mean, they're [private entities] obviously a big player in this because they are substantially driving the field if we think about all these health apps. So far, they are actually shaping some of the stuff that perhaps others should shape but it’s just because they're doing it and they're not asking too much too many questions... They just want to produce products... Nevertheless, we would certainly continue also to, you know, aim to work with them. You find some yeah enlightened groups there to work with [and] not get this feeling of you know exploitation in some ways or going on their own in the wrong direction this is really important* ...

[Participant 4]

Program directors also described difficulties in establishing a shared vocabulary and a common definition of “digital public health” among partners. Funding and resource challenges were frequently mentioned, with some programs relying heavily on grant funding to support their programs. These resource constraints influenced decisions regarding the depth versus breadth trade-offs in curriculum design and the need to compensate instructors for their time. In addition, resourcing challenges impacted decisions to limit the number of partnerships pursued for curriculum implementation.

Additional challenges included difficulties in gaining accreditation, particularly within traditional public health education systems that may undervalue the goal of developing T-shaped professionals. These challenges were addressed by securing leadership buy-in and institutional support. Program directors also emphasized the difficulty of creating curricula responsive to the rapid evolution of digital technology. To address this, they focused on fundamental principles such as systems thinking, privacy, and data governance, while incorporating applied training. This approach ensures students interact with current tools to better understand and apply these foundational principles.

## Discussion

### Principal Findings

In this environmental scan of DPH programs, we identified 58 programs and courses on digital competencies worldwide, the majority of which were in high-income countries. These initiatives addressed digital competencies across 3 main areas. The first 2 were related to public health data science and public health informatics, which build competencies for digital data, data systems management, and governance, consistent with our previous research showing this as a focus for digital competencies in public health [12]. Furthermore, we identified a third category of DPH programs and courses leveraging varied digital content applied in health promotion, program development and evaluation, epidemiology, disease surveillance, and public health. This category also included programs and courses aimed at understanding and addressing the digital determinants of health. For the interdisciplinary programs, most have aligned with external pressures from job market trends, student demands, and curricular recommendations for interdisciplinary practice [15,16]. While most programs emphasize applied learning within interdisciplinary frameworks to develop T-shaped professionals, challenges such as funding constraints and difficulties in achieving shared public health commitments and vocabulary remain significant barriers.

To the best of our knowledge, this is one of the first global scans of DPH programs and courses. Previous jurisdiction-specific scans, such as one in Germany, indicated that only about 20% of public health programs integrate digital competencies across their curricula [29]. While the German scan highlighted a strong focus on digital data analytics and informatics, it noted limited attention to social and equity perspectives in DPH—findings consistent with our scan. However, our global scan identified a few programs beginning to address these social perspectives, including understanding and tackling the digital determinants of health [29,30]. In Canada, while no specific environmental scans have been conducted, literature reviews emphasize the need for competency updates to support the deployment of artificial intelligence and other DPH interventions in public health [12,31]. Our study extends the literature by characterizing the nature of inter- and transdisciplinary partnerships, which are critical for designing and delivering such training programs [17,29,32]. The geographic distribution of programs and courses we identified shows a concentration in high-income countries, highlighting significant digital competency gaps in low- and middle-income countries. In many low- and middle-income country contexts, limited digital competency within the public health workforce, often coupled with low self-efficacy, is a key barrier to the uptake of digital technologies [33,34]. This is a gap requiring adaptive models that can leverage open-access curricula and a blend of learning approaches that are adapted to resource-constrained settings.

Given global recognition of the public health implications of societal digital transformation, our study offers valuable guidance to update public health workforce training programs [8]. In Canada, the Chief Public Health Officer’s vision for transforming public health prioritizes the use of digital technology [13]. Our findings show that schools of public health are adapting to this global vision in nonstandardized ways. They have focused primarily on data-centric fields such as data science and public health informatics for surveillance, epidemiology, and research through biostatistics and computer sciences [12,29,31]. Furthermore, few programs have comprehensively addressed broader DPH issues, such as digital health promotion, program evaluation, leadership, new media communications, and the digital determinants of health [12,29,35]. Contemporary challenges, such as mis- and disinformation exacerbated by digital transformation, underscore the need to expand efforts in these areas [32,35-37].

**Figure 2.**
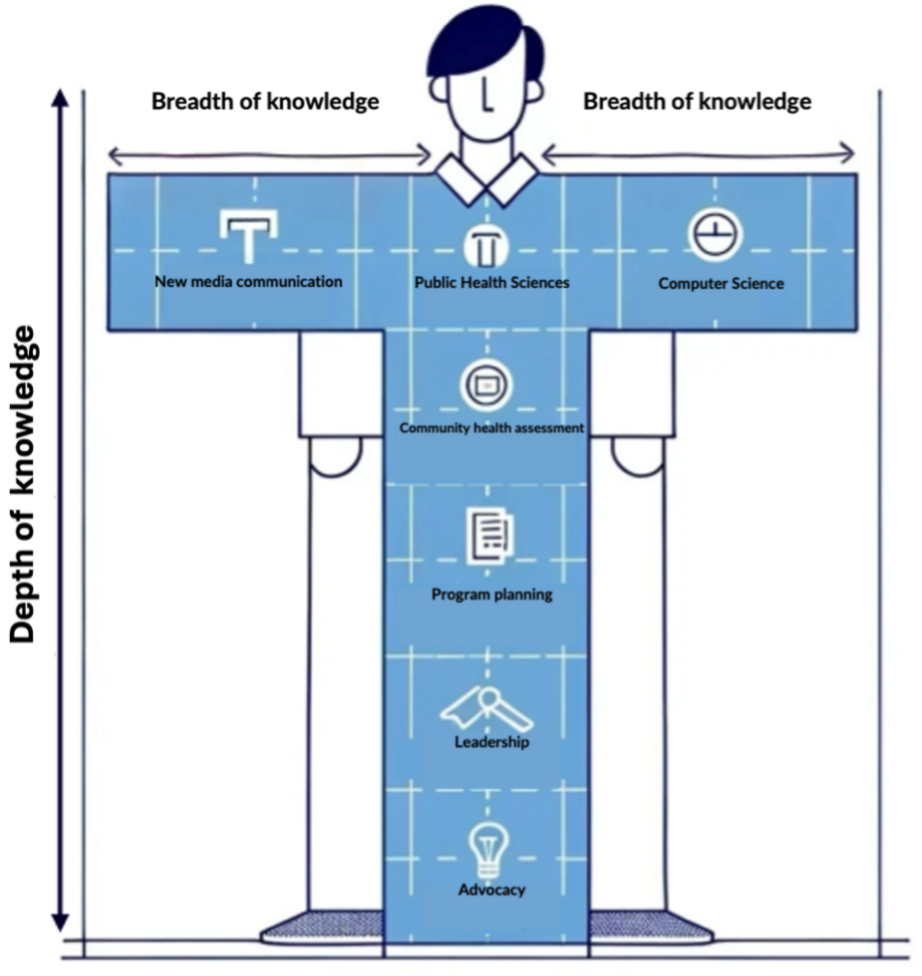
Depiction of a T-shaped professional.

While the ASPHER task force on digital transformation has recommended integrating digital determinants of health into curricula, their suggestion to avoid stand-alone courses does not fully support the development of inter- and transdisciplinary skills required for specialized DPH roles [16,38]. Public health professionals require both foundational knowledge through curricular integration and deeper expertise through stand-alone programs or courses [38]. A stepwise approach is needed to review and integrate digital competencies into existing public health curricula as core competencies for all professionals, while also offering specialized training opportunities [38]. This approach must prioritize inter- and transdisciplinary frameworks to develop “T-shaped professionals” equipped to navigate complex challenges while ensuring opportunities for applied training in real-world contexts (Figure 2) [38-40].

However, adopting this inter- and transdisciplinary paradigm requires addressing challenges such as partnerships, administrative barriers, and funding constraints within schools of public health [40,41]. Funding is essential for baseline integration and the design of specialized DPH programs. Advocacy and collaboration with organizations aligned with transdisciplinary public health goals can catalyze this process, as has been demonstrated by programs such as the Canadian Health Systems Impact Fellowship [42]. Establishing transdisciplinary programs requires engaging motivated partners first, gradually expanding to include other collaborators with clearly defined roles and expectations based on desired graduate competencies [39-41]. Engagement with public and private health sectors and digital actors must follow a principles-driven approach that prioritizes equity and health over commercialization and techno-optimism [9,43].

Reflexivity, ongoing dialog, and program co-creation with partners are essential for the effective development and implementation of these programs. Public sector collaboration is crucial to ensuring programs align with health system needs. Educators must clearly define the value and roles of program graduates to enhance their real-world impact. In addition, curricula should prioritize evaluation using clear indicators to measure effects on partner organizations, workforce competencies, and public health outcomes. As many of the identified programs are relatively new, formal evaluations were considered beyond the scope of this study. We recognize that assessing outcomes will be essential for understanding their effectiveness and for securing long-term funding and institutional support [41].

### Strengths and Limitations

The findings from this study should be interpreted considering its strengths and limitations. To our knowledge, this is the first systematic documentation of DPH training programs globally. Our approach combined a review of available programs with interviews with training program staff, ensuring both rigor and a deeper understanding of program contexts. We also aimed for an expansive review by including all eligible programs worldwide. However, our reliance on Google Search and the use of English-only terms in the search strategy may have introduced bias, as search algorithms are location specific and prioritize popular programs. To mitigate this, we used Google Translate where appropriate to ensure returns in languages other than English were not excluded. In addition, this study did not comprehensively review all nondigital courses in public health training programs to assess whether current coursework incorporates DPH perspectives. Other similar environmental scans adopted this approach and showed approximately 20% of programs have considered DPH mainly using similar approaches as identified in our review [29]. Our assumptions of digital programs and courses being hosted within the schools of public health, as demonstrated in our search, may have inadvertently missed important DPH programs hosted in other allied schools or programs leveraging cross-listed courses. We also acknowledge limitations in the sample size for the interview with only 4 participants responding out of 22 invitations sent. This limited us from making any interjurisdictional comparisons. While our coding was inductive and did not follow a predetermined framework, the limited sample size led to categories that closely mirrored the main areas outlined in the topic guide. Nevertheless, the breadth and depth of conversations in the interviews ensure that we can gain useful insights from other programs while encouraging careful appraisal of readers’ own context when considering transferability of findings. Further research could include more broad qualitative inquiry of international perspectives, but our work represents an important first step.

### Conclusions

This environmental scan of DPH training programs highlights the predominance of data-centric competencies, such as public health data science and informatics, while identifying critical gaps in addressing competencies for digital health promotion, program evaluation, leadership, and digital determinants of health. Despite efforts by schools of public health to align with global and national priorities, progress remains fragmented and geographically limited. To address these gaps, we recommend a stepwise approach that integrates digital competencies as core elements in public health curricula while developing stand-alone programs to cultivate specialized skills. Emphasizing inter- and transdisciplinary frameworks is essential for building “T-shaped professionals” capable of navigating complex public health challenges, but overcoming barriers related to funding, administrative constraints, and partnerships will require sustained advocacy, principles-driven collaboration, and robust evaluation frameworks. These efforts must prioritize equity and health outcomes over commercial imperatives, ensuring that DPH training equips the workforce to respond effectively to the challenges and opportunities of societal digital transformation.

## Supplementary material

10.2196/73088 Multimedia Appendix 1 List of countries included in Google advanced search.

10.2196/73088 Multimedia Appendix 2 Data extraction form for environmental scan.

10.2196/73088 Multimedia Appendix 3 Interview guide for semistructured interviews.

10.2196/73088 Multimedia Appendix 4 Consent form for interviewers.

10.2196/73088 Multimedia Appendix 5 Summary of all included programs.

## Abbreviations

ASPHER: Association of Schools for Public Health in the European Region
DPH: digital public health
