# Supplementary material for: Building the Workforce’s Capacity to Support the Digital Transformation of Public Health: Environmental Scan of Training Programs for Digital Technologies in Public Health
Source: JMIR Public Health Surveill. 2025 Oct 15;11:e73088. doi: 10.2196/73088 (PMC12527317; doi:10.2196/73088)
Supplement: Multimedia Appendix 1 [file publichealth-v11-e73088-s001.docx]

## *Appendix 1 – Countries including in Google advanced search.*

| **S/N** | **Country**^[[1]](#footnote-1)^ |
| --- | --- |
| 1 | United states of America |
| 2 | Canada |
| 3 | United Kingdom |
| 4 | Australia |
| 5 | France |
| 6 | Italy |
| 7 | Sweden^[[2]](#footnote-2)^ |
| 8 | China |
| 9 | Switzerland |
| 10 | Germany^[[3]](#footnote-3)^ |

1. United States, Canada, United Kingdom, Australia, France, and Italy have been highest contributor to previous reviews of digital technologies for public health including (Iyamu, I., Xu, A. X. T., Gómez-Ramírez, O., Ablona, A., Chang, H. J., Mckee, G., & Gilbert, M. (2021). Defining Digital Public Health and the Role of Digitization, Digitalization, and Digital Transformation: Scoping Review. JMIR public health and surveillance, 7(11), e30399. <https://doi.org/10.2196/30399>; Iyamu, I., Gómez-Ramírez, O., Xu, A. X., Chang, H. J., Watt, S., Mckee, G., & Gilbert, M. (2022). Challenges in the development of digital public health interventions and mapped solutions: Findings from a scoping review. Digital health, 8, 20552076221102255. https://doi.org/10.1177/20552076221102255) [↑](#footnote-ref-1)
2. Sweden demonstrated to integrate digital technologies in public health in various public health domains including health prevention (Rapid Response Service. A review of internet-based testing services for HIV and sexually transmitted infections (STIs). Toronto, ON: The Ontario HIV Treatment Network; March 2022.) [↑](#footnote-ref-2)
3. Germany found to have schools for digital technologies in public health and have explored digital public health in significant detail (Darmann-Finck, I., Rothgang, H., & Zeeb, H. (2020). Digitalisierung und Gesundheitswissenschaften – White Paper Digital Public Health (Digitalization and Health Sciences - White Paper Digital Public Health). Gesundheitswesen (Bundesverband der Arzte des Offentlichen Gesundheitsdienstes (Germany)), 82(7), 620–622. https://doi.org/10.1055/a-1191-4344) [↑](#footnote-ref-3)
