# Supplementary material for: Building the Workforce’s Capacity to Support the Digital Transformation of Public Health: Environmental Scan of Training Programs for Digital Technologies in Public Health
Source: JMIR Public Health Surveill. 2025 Oct 15;11:e73088. doi: 10.2196/73088 (PMC12527317; doi:10.2196/73088)
Supplement: Multimedia Appendix 2 [file publichealth-v11-e73088-s002.docx]

## *Appendix 2 - Data Extraction Form for environmental scan (blank)*

**Program curricula and websites**

**Environmental Scan Data Extraction Form**

| ID no. (as assigned in Covidence) |  |
| --- | --- |
| Name of the program: |  |
| Country of program: |  |
| City of program (if stated. More important for Canadian programs) |  |
| Year of commencement of program (if available): |  |
| Data extraction completed by: |  |

**Summary Notes**

|  |
| --- |

**Program Description**

| **Theory:** Describe the educational theory(ies), concept or training approach used in this program |  |
| --- | --- |
| **Learning objectives:** Describe the learning objectives for all groups involved in the program |  |
| **Target audience:** Describe the audience the program is targeted at. |  |
| **Level of training:** (i.e., undergraduate, masters, doctoral level and professional certificates) indicate all that apply |  |
| Funding reported (if any) |  |
| Partners (i.e., public and private sector partners reported (if any)). |  |
| **Materials:** Describe the educational materials used in the program. |  |
| **Materials:** Describe the technologies are focused on in the training program. Indicate all that apply. |  |
| **Materials:** Describe the public health domains considered in the training programs. Indicate all that apply. |  |
| **Educational strategies:** Describe the teaching and learning strategies used (e.g., tutorials, lectures, online modules, on-the-job training/internships) |  |
| **Instructors:** Describe the professional disciplines involved in the design, delivery and evaluation (if any) of the training program. |  |
| **Instructors:** (If more than one professional discipline involved) Describe the type of collaborations and extent of the collaborations. |  |
| **Delivery:** Descriptve the modes of delivery of course materials (e.g. face-to-face, internet or independent study package) of the educational intervention. Is the material provided individually or in a group and what is the ratio of learners to instructors? |  |
| **Environment:** Describe the physical or virtual spaces where learning occurred. |  |
| **Duration** of training program (in years) |  |
| **Changes:** Are there any changes reported by the program in response to students’ needs or to the context? |  |
