# Supplementary material for: Building the Workforce’s Capacity to Support the Digital Transformation of Public Health: Environmental Scan of Training Programs for Digital Technologies in Public Health
Source: JMIR Public Health Surveill. 2025 Oct 15;11:e73088. doi: 10.2196/73088 (PMC12527317; doi:10.2196/73088)
Supplement: Multimedia Appendix 3 [file publichealth-v11-e73088-s003.docx]

## *Appendix 3 – Preliminary Interview Guide for Semi-Structured Interviews*

Date: _____________ Time started: __________Time ended: __________Interview by: ____________

1. **Warm up:** How long have you worked at this institution?
2. **Warm up:** Can you tell me about your role in this program/course?
3. Can you tell me a little more about the program/course?
   1. When was it design and launched?
   2. Why was the program/course launched? What need was the program/course designed to meet?
   3. What competencies is the program/course designed to address?
   4. Who was involved in the design of the program/course?
   5. Who is the ideal student for the program/course?
   6. What would a student expect if they participated in the program/course?
   7. What are the professional qualifications of the course instructors? What professional disciplines are involved? If more than one discipline is involved, can you please describe the way the various disciplines work to design and deliver the program/course?
   8. What public health domains are focused on in the training? Any reason(s) why specific domains were focused on?
4. How is the program curriculum delivered?
   1. Is the course material provided online or through in-person contact?
   2. What additional methods of teaching are employed? E.g., internships or on-the-job training?
5. How would you describe the collaborations between professional disciplines involved in the design and delivery of the intervention?
   1. What has worked well with these collaborations?
   2. What has not worked will with these collaborations?
6. How would you describe the jobs that program graduates go on to do?
7. Are there additional program documents that you would like to share with us?

**Biodata**

Name of program represented: _______________City: ____________

Country: _____________________ Position in the program: _____________

Years in the position ____________________________
