# Supplementary material for: Building the Workforce’s Capacity to Support the Digital Transformation of Public Health: Environmental Scan of Training Programs for Digital Technologies in Public Health
Source: JMIR Public Health Surveill. 2025 Oct 15;11:e73088. doi: 10.2196/73088 (PMC12527317; doi:10.2196/73088)
Supplement: Multimedia Appendix 4 [file publichealth-v11-e73088-s004.pdf]

UBC Centre for Disease Control
655 West 12th Avenue
Vancouver, BC, Canada V5Z 4R4
Phone 604 707 2400
Fax 604 707 2401
www .bccdc.ca

CONSENT FORM (for interviews)

Fostering workforce capacity to maximize opportunities for the digital transformation of public health in Canada

Who is conducting the study?

Principal Investigator: Mark Gilbert, MD, MSc, Public Health Physician, Clinical Prevention Services, British Columbia Centre for Disease Control (BCCDC) & Associate Professor, School of Population and Public Health (SPPH), University of British Columbia (UBC). Phone: 604-707-5619; Email: mark.gilbert@bccdc.ca

Why are we doing this study?

The goal of this study is to enhance Canada's workforce capacity to maximize opportunities for the digital transformation of public health. More specifically, we aim to identify recommendations on competencies relevant in digital public health practice, training components that update our public health workforce capacity, and partnerships needed to implement these training components.

The rapid deployment of digital tools and their attendant successes and failures during the COVID-19 pandemic drew attention to the critical gaps the public health workforce's capacity to maximize the potential of digital technologies. To address this gap, we seek to discuss with Canadian public health trainers and experts to know what skills and training are needed for people working in public health, how the gaps in our Canadian training programs can be filled and what partners will be needed to do so. Findings from the study will help us propose new skills, training and partners that can be included in Canada's current training programs.

Who can participate in this study?

- Are you a staff or faculty in a public health training program?
- Have you been involved in designing program materials including program websites, published curricula and course syllabi, related to public health training programs.

If you have answered "Yes" to the questions above and if you are able to communicate in English, you may be eligible to participate

How is this study done?

This study employs a rapid literature review, an environmental scan, and focus groups. You are being asked to participate in one of our study interviews for the environmental scan.
The interview will be held virtually on Zoom. The interview will involve a review of currently existing program materials including program websites, published curricula and course syllabi, related to public health training programs.

Each interview is expected to take around 60 minutes to complete. We will record the interview, through the Zoom's "record" feature (on UBC's zoom license). You may or may not turn on your Zoom video, at your convenience. We will only be using the audio recordings for our study; any video recording files will be discarded immediately after the interview. Recording the interview sessions allows the interviewer(s) to concentrate on listening attentively to what is being said, and to transcribe and more accurately present the insights you share. We will also turn on the live transcription using Zoom's closed captions feature during the interview. You will only talk about information that you feel comfortable sharing with us. The interviewer(s) will take brief notes during the sessions, and, with your permission, ask follow-up questions after the session, if any. Your name will not be attached to any of these notes. The audio recordings will be transcribed, and the transcripts will be de-identified.

Who is funding this study?

This research is funded by the Canadian Institutes of Health Research (CIHR).

What are the potential harms of participating?

We do not think there is anything in this study that could harm or be bad for you. All participants will be asked not to repeat what is said in the interview anyone. We will also remove names, other potentially unique identifiers, and sensitive information from any reports of the completed study. Please let one of the study investigators know if you have any concerns. You do not have to answer any questions if you do not want to.

What are the potential benefits of participating?

Taking part in this study may or may not benefit you individually. However, your participation will help build capacity of the broader public health academia and implementers in maximizing digital technologies.

How will your confidentiality be protected?

Your confidentiality will be respected. Information that discloses your identity will not be released without your consent unless required by law. Your name will not be included in any of the reports of the completed study. We will also take steps to remove other sensitive information that could be used to identify you from any reports of the completed study. Recordings, transcripts and notes from the interview, and electronic records, including copies of consent forms and other documents you share with us, will be password-protected and encrypted, stored on a secure network drive of the UBCCDC/BCCDC Data Repository.

Additionally, research data may be required to be made publicly available to publish the insights gained from this study. In such case, only de-identified aggregate data would be stored in a public data repository. Once the data is made publicly available, we will not be able to withdraw your data.

What happens with the results?

Results obtained through this study will help us propose new skills, strategies, training competencies and plan partnerships that can be included in our current training programs in Canada.

The research findings will be made publicly available. This may include publishing in academic publications (in peer-reviewed journals, edited volumes, books, etc.), posting to a website, presenting at conferences and other events (e.g., classes, community forums, workshops, etc.), and other forms of public records (e.g., reports, policy briefs, blog entries, infographics, posters, press releases, organizational process maps, etc.) or sharing with research participants and others during a community event. The insights learned from this study will be used for knowledge translation, dissemination, and synthesis.

Will you be paid for your time?

There is no monetary compensation for your time to do this interview.

Is participation voluntary?

Participation is completely voluntary; you are under no obligation to participate. If you choose to participate, you may refuse to answer any questions. You indicate your consent to participate in the study by returning a signed consent form. A digital or electronic signature, or a scanned copy of a signed paper form is accepted.
If you change your mind about participating, you may simply inform our research assistant Swathi Ramachandran, of your desire to withdraw by e-mail to dishiresearch@bccdc.ca. You can withdraw your participation at any time during or after the interview, until the results have been made publicly available. We will not be able to honor your decision to withdraw, if the results have already been made publicly available. If for any reason you decide to withdraw your consent to participate, all data obtained through your interview will be discarded.

Who can you contact if you have questions about the study?

If you have any questions about the study, please contact Dr. Swathi Ramachandran (research assistant) by e-mail to dishiresearch@bccdc.ca.

Who can you contact if you have complaints or concerns about the study?

If you have any concerns or complaints about your rights as a research participant and/or your experiences while participating in this study, contact the Research Participant Complaint Line in the UBC Office of Research Ethics at 604-822-8598 or if long distance e-mail RSIL@ors.ubc.ca or call toll free 1-877-822-8598.

Consent and signature

Taking part in this study is entirely up to you. You have the right to refuse to participate in this study.

If you decide to take part, you may choose to pull out of the study at any time without giving a reason and without any negative impact on your employment or social standing.

• Your signature below indicates that you have received a copy of this consent form for your own records.

• Your signature indicates that you consent to participate in this study.

___________________________	___________________________	______________________
Participant's Name (please print)	Signature	Date

Do you agree to be contacted to ask you follow-up or clarifying questions if any?	☐ YES	☐ NO
Would you like to be contacted to provide input on knowledge translation plans?	☐ YES	☐ NO
Would you like to receive an update about the results of this study once it has been completed?	☐ YES	☐ NO

If yes to any of the questions above, please provide an email address: ______________________________
