# Supplementary material for: Building the Workforce’s Capacity to Support the Digital Transformation of Public Health: Environmental Scan of Training Programs for Digital Technologies in Public Health
Source: JMIR Public Health Surveill. 2025 Oct 15;11:e73088. doi: 10.2196/73088 (PMC12527317; doi:10.2196/73088)
Supplement: Multimedia Appendix 5 [file publichealth-v11-e73088-s005.docx]

*Appendix 4 – Summary of all included programs*

| **Serial Number** | **Name of the program, University:** | **Country of program:** | **Category*** | **Level of training:** (i.e., undergraduate, masters, doctoral level and professional certificates) indicate all that apply | **Materials:** Describe the public health domains considered in the training programs. Indicate all that apply. | **Instructors:** Describe the professional disciplines involved in the design, delivery and evaluation (if any) of the training program. | **Delivery** | **Duration** of training program (in years) | **Contacted for interview?** |
| --- | --- | --- | --- | --- | --- | --- | --- | --- | --- |
| 1 | Master of Science in Public Health Data Science, Boston University[1] | United States | Public health Data Science/data analytics | Masters | Epidemiology, climate and health, global health and implementation and translational sciences | NS | Blended | 1 year full-time or 2-years part time |  |
| 2 | Master of Science in Public Health - Public Health Data Science (PHDS) track, Columbia University[2] | United States | Public health Data Science/data analytics | Masters | Health research | Biostatistics and Epidemiology | In-person with applied sections e.g., internship or practicum | 2 years |  |
| 3 | Master of Science in Health Data Science, Harvard university[3] | United States | Public health Data Science/data analytics | Masters | Statistics, computing and public health sciences | Statistics, computing and public health sciences | In-person | 16 months | Yes |
| 4 | Digital and Mobile Health Research in Public Mental Health - Johns Hopkins Bloomberg School of Public Health[4] | United States | Mixed bag | Graduate level | Health research, Health promotion, program development and evaluation | NS | Online | 3 months (offered as part of graduate level public health programs) |  |
| 5 | Health Management Information Systems - Johns Hopkins Bloomberg School of Public Health[5] | United States | Public health informatics/information management | Masters and DrPH | Health research | Course taught by an industry leader working in an applied field | Blended | 3 months (offered as part of graduate level public health programs) |  |
| 6 | Master of Science in Public Health Data Science, Temple University[6] | United States | Public health Data Science/data analytics | Masters | Health research | NS | In-person | 16 months |  |
| 7 | Master's Degree in Health Data Science, University of California San Francisco[7] | United States | Public health Data Science/data analytics | Masters | Health research | States that it is largely influenced by data science, biostatistics and public health | In-person with applied sections e.g., internship or practicum | 2 years |  |
| 8 | Master of Science in Health Data Analytics, University of Louisville[8] | United States | Public health Data Science/data analytics | Masters | Health research | Public Health, Statistics | Online | 1.5 years |  |
| 9 | Master of Science in Health Data Science, University of Michigan School of Public Health- Ann Arbor[9] | United States | Public health Data Science/data analytics | Masters | Biostatistics and Health. Research | NS | In-person | 2 years |  |
| 10 | Public Health Data Science MPH, University of Minnesota[10] | United States | Public health Data Science/data analytics | Masters | Health research | Biostatistics and other faculty in the school of Public Health - not clear if there are partnerships outside the school | Blended | NS - 43 credits suggest 2-year training | Yes |
| 11 | Master of science in Public Health Data Science, Keck School of Medicine of USC[11] | United States | Public health Data Science/data analytics | Masters | Health research | Biostatistics, epidemiology, and computer science | In-person | 4 semesters |  |
| 12 | Master of Science in Biostatistics and Data Science (MSBDS), Washington University School of Medicine - Institute for informatics, data science and biostatistics[12] | United States | Public health Data Science/data analytics | Masters | Health research, Epidemiology | Statistics, public health and computer science | NS | 18 months - full time or up to 3 years part time |  |
| 13 | HLTH 473 - Digital Health, University of Waterloo - School of Public Health sciences[13] | Canada | Mixed bag | Undergraduate | Public health surveillance | NS | NS | 3 months (offered as part of undergraduate level public health programs) |  |
| 14 | Master of Health Informatics, University of Waterloo - School of Public Health sciences[14] | Canada | Public health informatics/information management | Masters | Not clearly stated - more public health generally | NS | Online with applied sections e.g., practicum | 16 months full time or up to 4 years part time |  |
| 15 | Dual Degree Program in Public Health Data Science (with university of Bordeaux), McGill University[15] | Canada | Public health Data Science/data analytics | Master’s and PhD | Health research | Epidemiology, statistics, informatics, Politics, Mathematics and Informatics and Life and Health sciences, communication, health economics, law, society, psychology, anthropology, management | In-person with applied sections e.g., internship or practicum | Masters - 1 yr, PhD - 3 years | Yes |
| 16 | Masters in Health data analytics and machine learning, Imperial University[16] | United Kingdom | Public health Data Science/data analytics | Masters | Public health generally | International experts in the data analytics field with real-world experience | Blended | 1 year | Yes |
| 17 | Master of digital health, La Trobe University[17] | Australia | Mixed bag | Masters | Epidemiology and public health | Disciplines include biostatistics, epidemiology and public health generally. Course was designed in consultation with industry partners | Blended | 2 years full time | Yes |
| 18 | Digital Health – Principles, Practice and Evidence (PHCM9790), University of New South Wales[18] | Australia | Mixed bag | Masters | Public health generally | Only one professor - a director at the WHO collaborating centre on digital health | Online | 1 term (3 months) |  |
| 19 | Master of public health data science, University of Bordeaux[19] | France | Public health Data Science/data analytics | Master’s and PhD | Surveillance, epidemiology, research | Describes multidisciplinary nature of the programs but does not clearly describe - Multidisciplinary training involving the teaching teams of the Bordeaux School of Public Health as well as French and international researchers. | Blended | 2 years | Yes |
| 20 | PhD in health data sciences, University of Berlin[20] | Germany | Public health Data Science/data analytics | Doctoral | Public health generally | The HDS PhD program is run by three main departments within the Charité/BIH: the Institute of Public Health (IPH), the Institute of Biometry and Clinical Epidemiology (iBikE) and the BIH Center for Transforming Biomedical Research (QUEST). | In-person | 3 years (6 semesters) | Yes |
| 21 | Biomedical Informatics - Master of Public Health, The Ohio State University[21] | United States | Public health informatics/information management | Masters | Public health generally | NS | NS | 2 years full time, 3 years part-time |  |
| 22 | Biostatistics and Health Analytics, M.S, St. Louis University[22] | United States | Public health Data Science/data analytics | Masters | NS - Public health generally | Partners with Biostatistics Consulting Service (BCS) - A consulting service advising on data analysis and appropriate methods of data presentation for publications, and provides design recommendations for public health and clinical research, including preparation of grant proposals | In-person | 2 years | Yes |
| 23 | MS - population health informatics, City University of New York School of Public Health[23] | United States | Public health informatics/information management | Masters | NS - Public health generally | NS | Online | 2 years/ 3 terms if full time. Not specified for Part time |  |
| 24 | Biostatistics, B.S. to Health Data Science, M.S. Accelerated Program, St. Louis University[24] | United States | Public health Data Science/data analytics | Bachelors and Masters | NS - Public health generally | Combined program | In-person | 5 years | Yes |
| 25 | Master of Science in Biostatistics with Concentration in Data Science in Public Health (MS), University of Memphis[25] | United States | Public health Data Science/data analytics | Masters | NS - Public health generally | Not specified, but includes computer science and mathematics more generally | In-person | 2 years, 4 terms |  |
| 26 | Bachelor of Science (BS) in Health Data Analytics, Drexel university Dornsife School of Public health[26] | United States | Public health Data Science/data analytics | Bachelors | NS - Public health generally | Reflecting the interdisciplinary approach of the Dornsife School of Public Health, students take courses in biostatistics, information science, public health, mathematics, epidemiology, and additional scientific disciplines of their choosing. | In-person | 4 years typically |  |
| 27 | Master of Health informatics, University of Michigan[27] | United States | Public health informatics/information management | Masters | Public health generally | NS | In-person | Typically 2 years |  |
| 28 | Executive MPH, Applied public health informatics, Emory university[28] | United States | Public health informatics/information management | Masters | Public health generally | NS | Blended - in-person sessions are optional | This 42-credit-hour degree program can be completed in two years (six semesters, completing three courses each semester) or three years (nine semesters, completing two courses each semester). |  |
| 29 | Public Health Communication and Marketing - MPH, George Washington University[29] | United States | Mixed bag | Masters | Public health generally; health promotion specifically | NS | Blended | 2 years full time |  |
| 30 | MS - Health analytics and biostatistics, University of Nevada[30] | United States | Public health Data Science/data analytics | Masters | Public health generally | Health informatics, Biostatics, Epidemiology in Public Health | In-person | 2 years full time |  |
| 31 | Public Health Informatics (Online NEW), Georgia Southern University[31] | United States | Public health informatics/information management | Masters | Public health generally | NS | Online | 2 years |  |
| 32 | MPH - public health data science, Gillings School of Global Public Health, University of North Carolina[32] | United States | Public health Data Science/data analytics | Masters | Public health generally | NS | Blended | NS |  |
| 33 | Master of Science in Computational Biology and Quantitative Genetics, Harvard University[33] | United States | Public health Data Science/data analytics | Masters | Public health surveillance | Modern molecular biology and genetics, computer programming | In-person | 18-24 months typically, for full time. Students may change to part time if necessary and take fewer than 15 credits per trerm. |  |
| 34 | MPH - health informatics, Augusta University[34] | United States | Public health informatics/information management | Masters | Public health generally | NS | Blended | Part-time and full-time schedules available that can be completed as soon as two years |  |
| 35 | MPH online - Health Information Management course, Morgan state University[35] | United States | Public health informatics/information management | Masters | NS - Public health generally | NS | Online | The Online MPH requires the completion of 42 credit hours: 35 hours of coursework and 7 credit hours to complete a graduate capstone project. The accelerated curriculum plan takes less than 18 months to complete with three full semesters and one summer semester. |  |
| 36 | MS Biostatistics - Statistical bioinformatics concentration, Northwestern University[36] | United States | Public health informatics/information management | Masters | Public health generally | NS | In-person | 1 year full-time or 2-years part time |  |
| 37 | MPH - Health informatics concentration, Case Western Reserve University[37] | United States | Public health informatics/information management | Masters | Public health generally | NS | In-person | as 16 months or up to 21 months depending on a student's status and course load. |  |
| 38 | MPH - Applied Epidemiology & Population Health Methods, University of San Francisco[38] | United States | Mixed bag | Masters | Epidemiology, biostatistics, public health surveillance and public health generally | rigorous training is paired with teaching through a social justice lens with a focus on reducing health disparities, promoting health equity, and improving population health, especially among historically and systemically disadvantaged communities. | Blended |  |  |
| 39 | The MS in Health Information Systems & Technology, Claremont Graduate University[39] | United States | Public health informatics/information management | Masters | NS | Health care administration and information technology | In-person | 2 years full time | Yes |
| 40 | MPH health analytics concentration, Stonybrook University[40] | United States | Mixed bag | Masters | NS - Public health generally | NS | In-person | 2 years |  |
| 41 | BSc Health sciences/Public health - Health informatics option, University of Waterloo[41] | Canada | Public health informatics/information management | Undergraduate | NS | Health Informatics, Information Technology | In-person | 4 years |  |
| 42 | MS, PhD in Biomedical Data Science, University of Wisconsin[42] | United States | Public health Data Science/data analytics | Master’s and PhD | Health research, Statistics | The program blends the best of statistics, computer sciences, biostatistics and biomedical informatics | In-person | 2 years for MS 5 years for PhD | Yes |
| 43 | Health Informatics & Data Science MS, Loyola University[43] | United States | Mixed bag | Masters | Health Informatics | NS | Online | 2 years full time and part time |  |
| 44 | MSc Spec-Course, part of Biostatistics, Dalla Lana School of Public Health, University of Toronto[44] | Canada | Public health Data Science/data analytics | Masters | Public health generally | Methods of Assessment Assignments (3 @ 20% each) 60% Final Exam 30% Participation 10% | In-person | 1 year |  |
| 45 | BSc and MSc Health and Informatics, University of Copenhagen[45] | Denmark | Public health informatics/information management | Masters | Informatics | Healthcare information technology, computer science | NS | 2 years |  |
| 46 | Masters in digital epidemiology, Manipal academy of higher education [46] | India | Mixed bag | Masters | Epidemiology. | Mathematics and/or Statistics and/or Computer Science | NS | 2 yearsfull time | Yes |
| 47 | Master of Science in Data Science, Manipal academy of higher education[47] | India | Public health Data Science/data analytics | Masters | Biostatistics and Health. Research | Biostatistics and Public Health | NS | 2 years full time | Yes |
| 48 | Public Health Informatics, University College Cork [48] | Ireland | Public health informatics/information management | Masters | Public Health information infrastructure | Public Health information infrastructure, influence of such communications; legal and ethical principles of communication; application of methods of communication | In-person | 1-year full time |  |
| 49 | Master of Computational Biology and Bioinformatics, New University of Lisbon [49] | Portugal | Public health informatics/information management | Masters | Informatics | Computer Science, Computational biology, bioinformatics | NS | 2 | Yes |
| 50 | Public health science: Digital health and communication - Master's program, University of Skövde [50] | Sweden | Mixed bag | Masters | Public health, health research, epidemiology, health communication | Leadership and health communication, Epidemiology and Scientific methods and ethical perspectives in public health science. | Online |  | Yes |
| 51 | Master of Health Data Science, London School of Hygiene and Tropical Medicine [51] | London | Public health Data Science/data analytics | Masters | Epidemiology and public health | Emerging discipline, combining mathematics, statistics, epidemiology and informatics | Blended with applied sections | One year full-time; part-time or split-study over two years | Yes |
| 52 | Public Health data science, University College London [52] | London | Public health Data Science/data analytics | Masters | Epidemiology and public health | Epidemiology, biostatistics, and public health | NS | 15 credits, 1 term? | Yes |
| 53 | Bachelor of Health Data Science, Richard Fairbanks School of Public Health - Indiana University–Purdue University Indianapolis [53] | United States | Public health Data Science/data analytics | Undergraduate | Public health, Informatics | Integrates biostatistics, computer science, and informatics | NS | 4 years | Yes |
| 54 | Master of Digital Public Health, Universiti Brunei Darussalam [54] | Brunei | Mixed bag | Masters | Public health generally | Epidemiology, biostatistics, and public health | NS | Full Time: 18-24 months  Part Time: 36-48 months | Yes |
| 55 | BA and MA study programs for digital public health, University of Siegen [55] | Germany | Mixed bag | Masters | Public health generally, Epidemiology |  | NS |  | Yes |
| 56 | Health and Digital Transformation- Master's program, Maastricht University [56] | Netherlands | Mixed bag | Masters | Public health - Health equity, Research, evaluation | Emphasizes on interdisciplinarity among health, data science, information technology | NS | 2 years | Yes |
| 57 | Collaborative Specialization in Machine Learning in Health and Biomedical Sciences, Western University [57] | Canada | Public health Data Science/data analytics | Master’s and PhD | Epidemiology | Biomedical Sciences, Computer Science, Epidemiology | Of parent program ie MSc in epi/biostat - in-person | 2 years for MSc |  |
| 58 | Artificial Intelligence (AI) for Epidemiological Studies Using UK Biobank Data, Oxford University [58] | United Kingdom | Public health Data Science/data analytics | Doctoral | Epidemiology, biostatistics, and public health; Cancer prevention | Epidemiology, biostatistics, and public health; Cancer prevention |  | 4 years typically |  |

NS – Not specified

* The categorizations are researchers’ interpretations.

**References**

1. Boston University school of public h. Master of Science in Public Health Data Science at Boston University. [Program of public health]; Available from: <https://www.bu.edu/sph/education/degrees-and-programs/ma-ms-programs/master-of-science-in-population-health-research-public-health-data-science/>.

2. Mailman School of Public health CU. Master of Science in Public Health - Public Health Data Science at Columbia University. Available from: <https://www.publichealth.columbia.edu/academics/degrees/degree-requirements/ms/public-health-data-science>.

3. T.H. Chan School of Public Health HU. Master of Science in Health Data Science at Harvard University. Available from: <https://content.sph.harvard.edu/biostats/publications/hds_handbook/hds_handbook.pdf>.

4. Johns Hopkins U. Digital and Mobile Health Research in Public Mental Health - Johns Hopkins Bloomberg School of Public Health. [Public health program]; Available from: <https://courseplus.jhu.edu/core/index.cfm/go/syl:syl.public.view/catalogID/36515>.

5. Johns Hopkins U. Health Management Information Systems - Johns Hopkins Bloomberg School of Public Health. Available from: <https://www.jhsph.edu/courses/course/35759/2022/312.633.81/health-management-information-systems>.

6. Temple University P. Master of Science in Public Health Data Science at Temple University. [Public health program]; Available from: <https://www.temple.edu/academics/degree-programs/public-health-data-science-ms-hp-phds-ms>.

7. University of California at San F. Master's Degree in Health Data Science at UC San Francisco. Available from: .

8. University of L. Master of Science in Health Data Analytics at University of Louisville. [MSc Program]; Available from: <https://louisville.edu/sphis/departments/hmss/academics-1/ms-in-health-data-analytic>.

9. University of Michigan School of Public Health- Ann A. Master of Science in Health Data Science at University of Michigan. Available from: <https://sph.umich.edu/biostat/programs/masters-hds.html>.

10. University of Minnesota School of Public h. MPH in Public health Data Science at University of Minnesota. Available from: <https://www.sph.umn.edu/academics/degrees-programs/mph/data-science/>.

11. University of Southern California KSoMUSCDoPh. Master of science in Public Health Data Science at University of Southern California. Available from: <https://keck.usc.edu/public-health-data-science-ms-program/>.

12. Institute for informatics ds, biostatistics WU. Master of Science in Biostatistics and Data Science at Washington University. Washington University School of Medicine - Institute for informatics, data science and biostatistics.

13. University of Waterloo School of Public health S. Digital Health at University of Waterloo. Available from: <https://uwaterloo.ca/public-health-sciences/current-undergraduate-students/department-consent-form-hlth-473-digital-health>.

14. University of Waterloo School of Public health S. Master of Health Informatics at University of Waterloo. Available from: <https://uwaterloo.ca/public-health-sciences/future-graduate-students/professional-programs/master-health-informatics>.

15. McGill University School of P, Global h. Dual Degree Program in Public Health Data Science at McGill university (with university of Bordeaux). Available from: <https://www.mcgill.ca/epi-biostat-occh/education/grad/dual-degree-programs-public-health-data-science>.

16. Imperial U. Masters in Health data analytics and machine learning. Available from: <https://www.imperial.ac.uk/study/courses/postgraduate-taught/health-data-analytics/>.

18. University of New South W. Digital Health – Principles, Practice and Evidence. Available from: <https://www.handbook.unsw.edu.au/postgraduate/courses/2020/PHCM9790>.

19. Bordeaux Uo. Master of public health data science, University of Bordeaux. Available from: <https://www.isped.u-bordeaux.fr/FORMATION/Formations-propos%C3%A9es/Les-Masters/Master-2-Public-Health-Data-Science-On-site>.

<https://cph.osu.edu/prospective-students/mph/biomedical-informatics>.

22. St Louis U. Master of Science Biostatistics and Health Analytics. [MS]; Available from: <https://www.slu.edu/public-health-social-justice/programs-and-certificates/health-analytics/>.

23. City University of New York SoPh. MS in Population Health Informatics. Available from: <https://sph.cuny.edu/academics/degrees-and-programs/masters-programs/ms-in-population-health-informatics/>.

24. Saint Louis U. Biostatistics, B.S. to Health Data Science, M.S. Accelerated Program. Available from: <https://www.slu.edu/public-health-social-justice/programs-and-certificates/health-analytics/biostatistics-bs-to-heath-data-science-ms-accelerated.php>.

25. University of M. Master of Science in Biostatistics with Concentration in Data Science in Public Health (MS). Available from: <https://www.memphis.edu/publichealth/programs/ms-biostatistics-dsph.php>.

26. Drexel university DSoPh. Bachelor of Science (BS) in Health Data Analytics. Available from: <https://drexel.edu/dornsife/academics/degrees/undergraduate-public-health-program/bs-health-data-analytics/>.

27. University of M. Master of Health informatics. Available from: <https://www.si.umich.edu/programs/master-health-informatics/curriculum>.

28. Emory U. Executive MPH, Applied public health informatics. Available from: <https://sph.emory.edu/departments/emph/index.html>.

29. George Washington U. Public Health Communication and Marketing - MPH. Available from: <https://publichealth.gwu.edu/programs/public-health-communication-and-marketing-mph>.

30. University of Reno N. MS - Health analytics and biostatistics. Available from: <https://www.unr.edu/public-health/degrees/ms-biostatistics/health-analytics-and-biostatistics-handbook>.

32. University of North Carolina at Chapel H. MPH - public health data science. Available from: <https://sph.unc.edu/resource-pages/master-of-public-health/>.

33. Harvard U. Master of Science in Computational Biology and Quantitative Genetics. Available from: <https://www.hsph.harvard.edu/sm-computational-biology/>.

34. Augusta U. Master of Public Health - health informatics. Available from: <https://www.augusta.edu/gradschool/mph>.

35. Morgan State U. Online MPH - Health Information Management. [OMPH]; Available from: <https://catalog.morgan.edu/preview_program.php?catoid=4&poid=1285&returnto=222>.

36. Northwestern U. MS Biostatistics - Statistical bioinformatics concentration. Available from: <https://www.feinberg.northwestern.edu/sites/biostatistics/curriculum/index.html>.

37. Case Western Reserve U. MPH - Health informatics concentration. Available from: <https://case.edu/medicine/pqhs/education/public-health/master-public-health/mph-curriculum/concentrations>.

38. University of San F. MPH - Applied Epidemiology & Population Health Methods. Available from: <https://www.usfca.edu/nursing/programs/graduate/public-health/program-overview/applied-epidemiology-population-health-methods>.

39. Claremont Graduate U. MS in Health Information Systems & Technology. Available from: <https://www.cgu.edu/academics/program/health-information-systems-technology/>.

40. Stonybrook U. MPH health analytics concentration. Available from: <https://publichealth.stonybrookmedicine.edu/academics/competencies>.

41. University of Waterloo SoPh. BSc Health sciences/Public health - Health informatics option. Available from: <https://uwaterloo.ca/public-health-sciences/current-undergraduate-students/majors-minors-specializations/health-informatics-option>.

42. University of Wisconsin at M. MS - Biomedical Data Science. Available from: <https://biostat.wiscweb.wisc.edu/education/current-students/ms-bds/>.

43. Loyola University C. Health Informatics & Data Science MS. Available from: <https://www.luc.edu/parkinson/academics/departments/healthinformaticsdatascience/mshealthinformatics/>.

44. University of Toronto DLSoPH. MSc: Biostatistics – Course-​only Option. Available from: <https://www.dlsph.utoronto.ca/programs/msc-biostatistics-course-only-option/>.

45. University of C. Master's Programme in Health and Informatics. Available from: <https://studier.ku.dk/kandidat/sundhed-og-informatik/>.

46. Manipal academy of higher e. Masters in digital epidemiology. Available from: <https://manipal.edu/psph/program-list/msc-digital-epidemiology.html>.

47. Manipal academy of higher e. Master of Science in Data Science. Available from: <https://manipal.edu/psph/program-list/msc-data-science.html>.

48. University College C. Master of Public Health (MPH) in public health informatics. Available from: <https://www.ucc.ie/en/ckx11/?gclid=EAIaIQobChMI18PLttmk4AIVTLHtCh2GZQbAEAAYASAAEgLmSPD_BwE>.

49. Universidade Nova de L. Master of Computational Biology and Bioinformatics. Available from: <https://www.ihmt.unl.pt/education/computational-biology-bioinformatics/>.

50. University of Skövde S. Master's - Public health science: Digital health and communication. Available from: <https://www.his.se/utbildning/halsa-och-vard/folkhalsovetenskap-digital-halsa-och-kommunikation-magisterprogram-fhdka/>.

51. London School of H, Tropical M. Master of Health Data Science. Available from: <https://www.lshtm.ac.uk/study/courses/masters-degrees/health-data-science>.

52. University College L. Public health data science - MSc module. Available from: <https://www.ucl.ac.uk/module-catalogue/modules/public-health-data-science-CHME0017>.

53. Indiana University Indianapolis RMFSoPH. Bachelor of Health Data Science. Available from: <https://fairbanks.indianapolis.iu.edu/academics/undergraduate/bshds/index.html>.

54. Universiti Brunei D. Master of Digital Public Health. Available from: <https://ubd.edu.bn/c3l/dph/master-digital-public-health/>.

55. <https://www.uni> sdzsmdhele. Digital Public Health - Master of Science. Available from: <https://www.uni-siegen.de/zsb/studienangebot/master/dph.html.en?lang=en>.

56. Maastricht U. Health and Digital Transformation - Master's programme. Available from: <https://curriculum.maastrichtuniversity.nl/education/master/master-digital-health-transformation/your-future>.

57. Western University C. Collaborative Specialization in Machine Learning in Health and Biomedical Sciences. Available from: <https://uwo.ca/sci/datascience/graduate/collaborative-specialization-ml-health-biomedical.html>.

58. University of O. Artificial Intelligence (AI) for Epidemiological Studies Using UK Biobank Data. Available from: <https://www.ndph.ox.ac.uk/study-with-us/dphil-population-health/dphil-research-projects/2022-dphil-projects/artificial-intelligence-ai-for-epidemiological-studies-using-uk-biobank-data>.
